# *De Novo* Genome Assembly and Annotation of *Leptosia nina* Provide New Insights into the Evolutionary Dynamics of Genes Involved in Host-Plant Adaptation of Pierinae Butterflies

**DOI:** 10.1093/gbe/evae105

**Published:** 2024-05-23

**Authors:** Yu Okamura, Heiko Vogel

**Affiliations:** Department of Biological Sciences, Graduate School of Science, The University of Tokyo, Tokyo 113-0033, Japan; Department of Insect Symbiosis, Max Planck Institute for Chemical Ecology, Hans-Knöll-Str. 8, Jena 07745, Germany; Department of Insect Symbiosis, Max Planck Institute for Chemical Ecology, Hans-Knöll-Str. 8, Jena 07745, Germany

**Keywords:** host-plant adaptation, herbivore, genome

## Abstract

In interactions between plants and herbivorous insects, the traits enabling phytophagous insects to overcome chemical defenses of their host plants have evolved multiple times. A prominent example of such adaptive key innovations in herbivorous insects is nitrile specifier proteins (NSPs) that enabled Pierinae butterflies to colonize Brassicales host plants that have a glucosinolate–myrosinase defense system. Although the evolutionary aspects of NSP-encoding genes have been studied in some Pierinae taxa (especially among *Pieris* butterflies), the ancestral evolutionary state of *NSP*s is unclear due to the limited genomic information available for species within Pierinae. Here, we generate a high-quality genome assembly and annotation of *Leptosia nina*, a member of a small tribe, Leptosiaini. *L. nina* uses as its main host Capparaceae plants, one of the ancestral hosts within Pierinae. By using ∼90-fold coverage of Oxford Nanopore long reads and Illumina short reads for subsequent polishing and error correction, we constructed a final genome assembly that consisted of 286 contigs with a total of 225.8 Mb and an N50 of 10.7 Mb. Genome annotation with transcriptome hints predicted 16,574 genes and covered 98.3% of BUSCO genes. A typical *NSP* gene is composed of three tandem domains found in Pierinae butterflies; unexpectedly, we found a new *NSP*-like gene in Pierinae composed of only two tandem domains. This newly found *NSP*-like gene in *L. nina* provides important insights into the evolutionary dynamics of domain and gene duplication events relating to host-plant adaptation in Pierinae butterflies.

**Significance**

To better understand the evolutionary history of chemical interactions between plant defenses and herbivore counter-adaptations, it is useful to investigate adaptive key innovations in herbivorous insects to their host plants. Larvae of Pierinae butterflies use gut-expressed nitrile specifier proteins (NSPs) to disarm the glucosinolate-based defense system of their Brassicales host plants. Here, we generated a high-quality genome assembly of a Pierinae species, *Leptosia nina*, which is a member of taxa within Pierinae, Leptosiaini. Unlike the well-studied *Pieris* species, which feeds on Brassicaceae hosts, *L. nina* feeds on Capparaceae, a plant family that is also a member of Brassicales. Previous *NSP*s were identified as having three tandem repeat domains; in our genome assembly, we found a *NSP*-related gene with only two domains. Because this two-domain *NSP* gene is potentially associated with insects’ use of Capparaceae as host plants, our findings shed new light on the evolutionary dynamics underlying Pierinae counter-adaptations and highlight the potential importance of both gain and loss of gene domains. The genome of *L. nina*—especially the molecular evolution of *NSP*-related genes—illustrates how Pierinae species adapt to the highly diverse glucosinolate-based defenses of their host plants.

## Introduction

How plants and herbivorous insects interact is crucial to the ecology of terrestrial ecosystems. In many of these interactions, traits enabling herbivore insects to overcome chemical defenses of plants have evolved multiple times (Ratzka et al. 2002; Li et al. 2003; Schramm et al. 2012; Krempl et al. 2016). Some of these adaptive mechanisms are considered evolutionary key innovations, because they allowed insect herbivores to colonize novel host plants and, subsequently, enabled them to diversify (Berenbaum et al. 1996; Wheat et al. 2007; Edger et al. 2015; Allio et al. 2021). Revealing the evolutionary dynamics of those key innovations in herbivores is central to understanding the evolution of chemical interaction between plants and herbivores (Futuyma and Agrawal 2009). However, the dynamics of those adaptive traits depend on knowledge about the molecular bases of the adaptation as well as about the genetic information of herbivorous insects, knowledge that is limited.

**Fig. 1.**
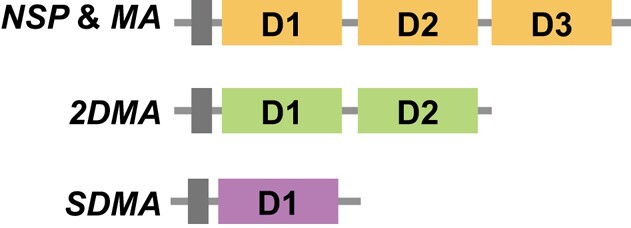
The domain coordination of genes in *NSP*-like gene family. Gray bars represent signal peptide regions, while colored boxes show repeat domain structures. Adapted from Fischer et al. (2008).

The nitrile specifier protein (NSP) gene and its sister gene, major allergen (MA) gene, are both found in Pierinae butterflies and are prominent examples of key innovations enabling herbivorous insects to adapt to their host plants (Wittstock et al. 2004; Wheat et al. 2007; Edger et al. 2015; Okamura et al. 2022). Because *NSP* and *MA* enable Pierinae butterflies to overcome the glucosinolate-based defense system in their Brassicales host plants (Fischer et al. 2008), the ecological relevance of these genes and proteins and their molecular evolutionary patterns have been extensively studied in some Pierinae taxa (Edger et al. 2015; Okamura et al. 2022). The family of *NSP*-like genes consists of domain-duplicated *NSP*, *MA*, and the single-domain major allergen (*SDMA*) (Fig. 1). *NSP* and *MA* genes are limited to Pierinae species that feed on Brassicales and are composed of three tandem repeat domains originating from *SDMA*; therefore, *NSP* and *MA* can also be considered as three-domain major allergens (*3DMA*s) (Fischer et al. 2008). While *SDMA* genes are broadly found in Insecta, *NDMA*s having multiple (N times) *SDMA* repeats encoding for a single polypeptide have also been observed (normally *2DMA*–*8DMA*) (Randall et al. 2013). However, in Lepidoptera, multi-domain *NDMA*s are so far limited to *NSP* and *MA* (*3DMAs*), and evolutionary dynamics within Pierinae remain unclear due to the sparse genomic data within Pierinae species (Fischer et al. 2008).

**Fig. 2.**
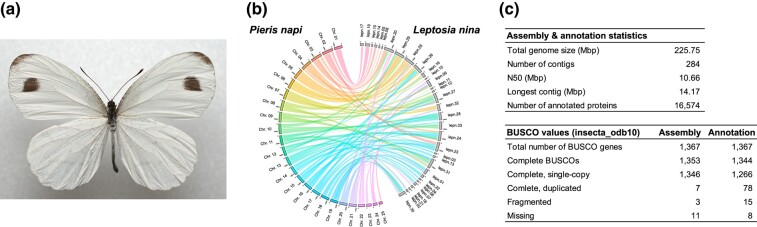
Assessment of assembled *L. nina* genome. a) *L. nina* adult male. b) Synteny plot between *L. nina* and *P. napi* genomes (colored). For the *L. nina* genome, only contigs larger than 1 Mb are shown, which includes 95.7% of the total assembled region. c) Statistics of the assembled *L. nina* genome and its gene annotation.

*Leptosia nina*, known as the wandering snowflake butterfly, is a member of Pierinae and uses Capparaceae, a glucosinolate-containing family in the order Brassicales, as its main host plant (Fig. 2a). *L. nina* is one of only two *Leptosia* species found in Asia; the other seven *Leptosia* species are mainly distributed in Africa. Although *Leptosia* is a small genus, it comprises a tribe, Leptosiaini, which, according to a dated Pierinae phylogeny (Edger et al. 2015), is distantly related to a well-studied and Brassicaceae-feeding tribe, Pierini (supplementary fig. S1, Supplementary Material online). To date, macroevolutionary dynamics of the *NSP*-like gene family have been tested mainly in Brassicaceae-feeding taxa (Edger et al. 2015; Okamura et al. 2019). Since Capparaceae feeding is considered an ancestral state to the Brassicaceae feeding (Edger et al. 2015), the genomic data from *L. nina* help clarify the origin and evolution of members of the *NSP*-like gene family in Pierinae.

Here, we generated a high-quality genome assembly of *L. nina* using Oxford Nanopore Technologies (ONT) long reads, Illumina short reads for error correction, and annotation and gene prediction based on RNA-seq data. Remarkably, we not only identified members of the *NSP* gene family in the genome but also confirmed the presence of another *NSP*-related gene in *L. nina*, which lost one domain compared to previously identified *NSP*s in Pierinae. The genome of *L. nina* may help answer both how *NSP*-related genes evolved in the Pierinae butterfly and whether these are associated with changes in the herbivore's host-plant repertoire.

## Results and Discussion

### Genome Sequence and Assembly Statistics

MinION long-read sequencing generated 21.5 Gb of data and an N50 of 7.1 kb, resulting in ∼90-fold genome coverage based on the estimated genome size of *L. nina* (∼250 Mb). We also generated 9.0 Gb of Illumina short reads for polishing and error correcting of the draft genome. The assembled haploid genome contained 284 contigs with a total genome size of 225.8 Mb and had 10.7Mb of N50 size. Assessment of the completeness of the genome assembly using BUSCO resulted in 99.0% BUSCO complete and single-copy orthologs and low duplication levels (0.5% duplicated BUSCO) (Fig. 2, supplementary fig. S2, Supplementary Material online). 87.4% of total RNA-seq reads were mapped to the assembled genome, and the BRAKER2 annotation pipeline with RNA-seq hint data annotated 16,574 genes with 98.3% complete BUSCO orthologs (92.6% single and 5.7% duplicated).

### Comparative Analyses

The assembled genome of *L. nina* was compared to the available chromosome-level genome assembly of one of the related species, *Pieris napi*. The synteny analyses showed a number of chromosome rearrangements between the two species (Fig. 2). The number of chromosomes of *L. nina*—*n* = 19 (Maeki and Ae 1968)—is smaller than that of *P. napi* with *n* = 25 (Hill et al. 2019). Although the two species are from the same Pierinae subfamily, the observed chromosome rearrangements between them indicate that such events occurred frequently in Pierinae. In addition, we also compared the *L. nina* genome with that of *Bombyx mori* (supplementary fig. S3, Supplementary Material online). Here, we confirmed that several chromosomes were rearranged but we also found syntenies in several chromosomes such as the sex chromosome.

### *NSP*-Related Genes

**Fig. 3.**
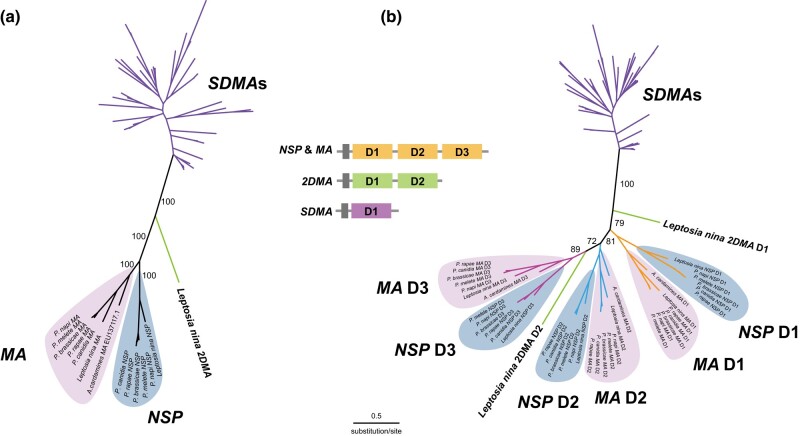
Phylogenetic relationships of *NSP*-like gene family members. a) Molecular phylogeny of the *NSP, MA*, *SDMA*, and *2DMA* genes based on the entire gene sequence (amino acid). b) Molecular phylogeny of the *NSP, MA*, *SDMA*, and *2DMA* genes based on the domain-level alignment (amino acid). The domain coordination of each gene is shown in the figure. Note *2DMA* domain 2 (D2) is located between the second and third domains of the *NSP* or *MA* genes in the tree.

In *L. nina* that fed primarily on glucosinolate-containing Capparaceae, we found both *NSP* and *MA* genes (Fig. 3a). Since Leptosiaini is relatively distantly related to the well-studied tribe, Pierini, the presence of both *NSP* and *MA* genes in this tribe shows that their acquisition occurred at an evolutionarily early stage of Pierinae diversification.

Unexpectedly, we also found a variant of *NSP* genes, namely *2DMA* in *L. nina*, which consists of two *SDMA* repeats encoding for a single polypeptide and has never been observed in any other Lepidopteran insect (Fig. 3a) (Randall et al. 2013). When screening other available Lepidopteran genomes in the NCBI database, we found several *2DMA*-like genes annotated, for instance, in *B. mori* and *Ostrinia furnacalis* (LOC101743760, LOC114364307). We found that those *2DMA*-like genes exhibit a high degree of sequence similarity between their first and second domains. Because the respective genomic sequences contained stop and start codons at the border of the two domains located in tandem, these supposedly *2DMA*-like genes were shown to result from mis-annotations of two tandem *SDMA*s. In *L. nina*, however, we confirmed the *2DMA* gene, based on the transcriptome assembly, gene prediction, as well as cDNA cloning and sequencing (supplementary fig. S4, Supplementary Material online, supplementary data S1, Supplementary Material online). No stop codon at the end of the first domain or a start codon at the beginning of the second domain was found, and the mRNA sequence was confirmed by cloning, indicating that the *2DMA* gene in *L. nina* encodes for a protein composed of two tandem domains.

The domain-level alignment analyses of *NSP*-related genes revealed that the first and second domains of *Leptosia 2DMA* had diverged (Fig. 3b). The first domain of *Leptosia 2DMA* had higher similarity to the first domain of *NSP* and *MA* genes, and the second domain of *Leptosia 2DMA* had higher similarity to the second and third domains of *NSP* and *MA* genes. There are two possible evolutionary scenarios for the emergence of *2DMA* in Pierinae butterflies: (i) *2DMA* originated from *NSP, MA*, or the ancestral sequences of these genes by the loss of its original second or third domain and was subsequently acquired by a subset of Pierinae species, including *L. nina*, and (ii) *2DMA* was first acquired among Pierinae butterflies as an ancestral state of *NSP* or *MA* genes, and the second domain was duplicated again, resulting in the formation of *NSP* or *MA* genes. Given the limitations of the current Pierinae genome datasets, it remains unclear which scenario is most likely and how the existence of *2DMA* might reflect patterns of host-plant specialization. Further analyses and data collection including Pierinae species from a broad taxon sampling are required to understand *2DMA* evolutionary dynamics among Pierinae butterflies. The selection analyses based on the domain-level alignment showed that dN/dS ratios of the two domains of *Leptosia 2DMA* were below one and not significantly different from those of *NSP*, *MA*, or *SDMA*. This suggests that it is less likely for *2DMA* to be a pseudogene (supplementary table S1, Supplementary Material online). Combining the observation of *2DMA* mRNA presence in the larval gut with this result potentially indicates that the functional role of *2DMA* could be related to the larval gut, similar to those observed for *NSP*, *MA*, and predicted for *SDMA*. Although confirmation of the presence of 2DMA protein in the larval gut and further functional analyses are still necessary, the identification of a *2DMA* gene in Capparaceae-feeding Pierinae species could be key to understanding the evolutionary dynamics of genes involved in Brassicales host-plant adaptation.

## Conclusions

In this study, we generate a high-quality genome assembly of *L. nina*, a member of a phylogenetically old tribe within Pierinae, Leptosiaini. Although *L. nina* feeds on glucosinolate-containing Brassicales plants, it specializes on Capparaceae, unlike the well-studied *Pieris* species that feed on Brassicaceae. Our genome assembly of *L. nina* had 225.8 Mb total genome size with an N50 of 10.7Mb and contained 99.0% of BUSCO genes. Significantly, within the genome assembly of *L. nina* and the larval transcriptome data, we discovered not only *NSP* and *MA*—two key genes in the Pierinae that facilitate their adaptation to glucosinolates in their host plants—but also another *NSP*-like gene that lacked one gene domain. Given the taxonomic significance and ecological distinctions of *L. nina* compared to other extensively studied Pierinae butterflies, these genomic data and the newly identified *NSP*-like gene provide crucial insights into understanding the co-evolutionary chemical arms race between Brassicales plants and Pierinae butterflies.

## Materials and Methods

### DNA Extraction and Sequencing

A male adult of *L. nina* was sampled at Yonaguni Island, Okinawa, Japan, in 2021 and stored in 100% ethanol at −20 °C until genomic DNA extraction. Genomic DNA was extracted using Nanobind Tissue Big DNA kit (Circulomics), after which Short Read Eliminator XS (Circulomics) was performed to selectively collect high-molecular-weight genomic DNA.

The isolated genomic DNA was used for library preparation for Oxford Nanopore MinION sequencing using Ligation Sequencing Kit (SQK-LSK109) and NEBNext® Ultra™ II End Repair/dA-Tailing Module. The prepared library was sequenced by one R 9.4.1 MinION flow cell. The MinION sequencing was done at the Max Planck Institute for Chemical Ecology, Jena, Germany. We performed base-calling using GUPPY v.4.0.11 (Wick et al. 2019) with a high-accuracy option (dna_r9.4.1_450bps_hac.cfg model) and generated 21.5 Gb of raw reads with N50 of 7.1Kb. The extracted genomic DNA from the same sample was also sequenced by Illumina HiSeq2500, and we acquired 9.2 Gb of high-accuracy short reads. The Illumina sequence was performed at the Max Planck Genome Center in Cologne.

### Assembly and Annotation

We assembled MinION long reads using two assemblers, namely Flye v.2.7 (Kolmogorov et al. 2019) and NECAT v.0.0.1 (Chen et al. 2021). The assembled genomes were both polished four times with Racon v.1.4.13 (Vaser et al. 2017) with (-m 8 -x -6 -g -8 -w 500) setting, once with Medaka v.1.0.3 (https://github.com/nanoporetech/medaka) with the r941_min_high_g344 model using the MinION raw reads, and, finally, with ntEdit v1.3.2 (Warren et al. 2019) using Illumina short reads. We performed PURGEhaplotigs v.1.0.3 (Roach et al. 2018) to purge diploid genomic regions and acquired haploid genome assemblies. The two polished haploid genomes from the two assemblers were then merged with quickmerge v0.3 (Chakraborty et al. 2016) using the Flye-generated genome as a template. The qualities of the assembled genomes were assessed by BUSCO with insecta_odb10 database (Seppey et al. 2019) and SeqKit v. 0.12.1 (Shen et al. 2016). The potential contaminants were checked by blobtools using UniProt and NR databases (Laetsch and Blaxter 2017).

The final polished genome assembly was used for genome annotation. To mask the repetitive region in the genome, we used Repeat Modeler v. 1.0.7 (Flynn et al. 2020), which implemented RECON v. 1.08 (Bao 2002); RepeatScout v. 1.0.5 (Price et al. 2005) and Tandem Repeats Finder (Benson 1999), which predicted the repeat structure; and Repeat Masker, which performed soft masking. We mapped RNA-seq reads (see below) with STAR v2.7 (Dobin et al. 2013) to the genome and annotated the soft-masked genome with BRAKER2 pipeline (Stanke et al. 2008, 2006; Hoff et al. 2019, 2016; Brůna et al. 2021) using RNA-seq mapping information as hints (Lomsadze et al. 2014). Functional annotation was performed using Blast2GO.

### Genome Synteny Analyses

We used NUCmer v. 3.1 (Kurtz et al. 2004) to align the *L. nina* genome assembly to the available chromosome-level genome assembly of *P. napi* (Hill et al. 2019), which is a closely related species. For visualization, we excluded contigs shorter than 1 Mbp from our *L. nina* genome. This process resulted in 33 contigs with 216 Mb total size keeping 95.7% of assembled genomic region.

### RNA Sequencing and *De Novo* Transcriptome Assembly

RNA was isolated from 5 *L. nina* larvae reared in the Ishikawa Insect Museum with *Crateva religiosa*. RNA was isolated using RNeasy Kits (QIAGEN). The quality of the RNA was assessed by an Agilent bioanalyzer, and a unit of RNA from five larvae was pooled for sequencing by Illumina at the Max Planck Genome Center in Cologne. We trimmed the raw reads using Trimmomatic with LEADING:10 TRAILING:10 SLIDINGWINDOW:4:20 MINLEN:40 option and de novo assembled the transcriptome using Trinity v. 2.1.1.

### NSP-Related Gene Analyses

We performed tblastn to search for potential *NSP-*related genes in the genome assembly, annotated *L. nina* gene sets, and de novo assembled transcriptome. We used published *NSP*-related gene sequences as queries including *NSP*, *MA*, and *SDMA* sequences from *Pieris brassicae*. The hits were aligned and trimmed using Mafft v. 7.487 (Katoh and Standley 2013) and MEGA7 (Kumar et al. 2016).

We performed molecular cloning to confirm the presence of *2DMA* mRNA in the gut-extracted RNA samples of *L. nina* larva. cDNA was synthesized by ReverTra Ace qPCR RT Master Mix with gDNA remover (TOYOBO), and Tks Gflex DNA polymerase (Takara) was used to amplify the full-length mRNA sequence of *2DMA* (primer F: ATGAAACTTATAATATTGTTGAGTTTTATA, primer R: TCATTCTTGACCAAAAATAGCCA). The PCR product was Gel-purified with NucleoSpin Gel and PCR Clean-up (Takara). For cloning, we used In-Fusion Snap Assembly Master Mix (Takara) and cloned the PCR products into pUC19 Vector. Several colonies were selected, and the insertion of the fragment (1.2 kb) was confirmed using colony PCR with EmeraldAmp MAX PCR Master Mix (Takara) and M13 primers. Colonies with proper plasmids were selected and incubated in 2 ml of Luria-Bertani (LB) medium with carbenicillin overnight. Plasmids were then extracted using NucleoSpin Plasmid Easy Pure (Takara). Purified plasmids were sequenced with M13 primers in Eurofin Genomics.

We annotated the domain structure based on a previous work and aligned the gene sequences at the level of both gene and domain. To evaluate the evolutionary relationships of the *NSP*-related genes, we performed phylogenetic analyses using the amino acid alignment of those *NSP*-related genes with IQtree v. 1.6.12 by using the auto-model finder as well as setting -bb 1000 -bnni for ultrafast bootstrap (Nguyen et al. 2015; Kalyaanamoorthy et al. 2017; Hoang et al. 2018). The model finder identified LG + F + G4 as the best model for the entire gene alignment and LG + I + G4 as the best model for the domain-level alignment, and an ML tree was estimated by IQtree based on these substitution models.

Based on the domain-level alignment, selection analyses were performed using CODEML implemented in PAML (Yang 2007). Two branch model tests were performed by setting the two *2DMA* domains (*L. nina 2DMA* D1 and *L. nina 2DMA* D2) as a foreground branch, respectively. In each test, a null model and an alternative model were run with setting (model = 0 and NSsite = 0) for a null model and (model = 2 and NSsite = 0) for an alternative model. Likelihood ratio tests were performed for comparing null and alternative models to see whether the selected foreground branches had significantly different dN/dS ratio compared to the background branches. In addition, we also calculated dN/dS for each terminal branch of genes from *L. nina* by setting model = 1 and NSsite = 0.

## Supplementary Material

evae105_Supplementary_Data

## Data Availability

The genome assembly and annotation data as well as the raw MinION reads, Illumina short reads, and RNA-seq reads have been deposited in ENA (PRJEB68217).
