# Supplementary material for: De Novo Genome Assembly and Annotation of Leptosia nina Provide New Insights into the Evolutionary Dynamics of Genes Involved in Host-Plant Adaptation of Pierinae Butterflies
Source: Genome Biol Evol. 2024 May 23;16(5):evae105. doi: 10.1093/gbe/evae105 (PMC11135640; doi:10.1093/gbe/evae105)
Supplement: evae105_Supplementary_Data [file evae105_supplementary_data.docx]

Supplementary figures for
***De novo* genome assembly and annotation of *Leptosia nina* provide new insights in the evolutionary dynamics of genes involved in host-plant adaptation of Pierinae butterflies**

Yu Okamura^1, 2^, Heiko Vogel^2^

1 Department of Biological Sciences, Graduate School of Science, University of Tokyo, Tokyo 113-0033, Japan

2 Department of Insect Symbiosis, Max Planck Institute for Chemical Ecology, Hans-Knöll-Str. 8, Jena, 07745, Germany

**Fig. S1 A** tribe phylogeny of Pierinae butterflies. The branch colors represent their primary host plant; yellow: Brassicaceae, green; Capparaceae or Cleomaceae, purple; Basal Brassicales, black, non-Brassicales. Adapted from Edger et al. (2015).


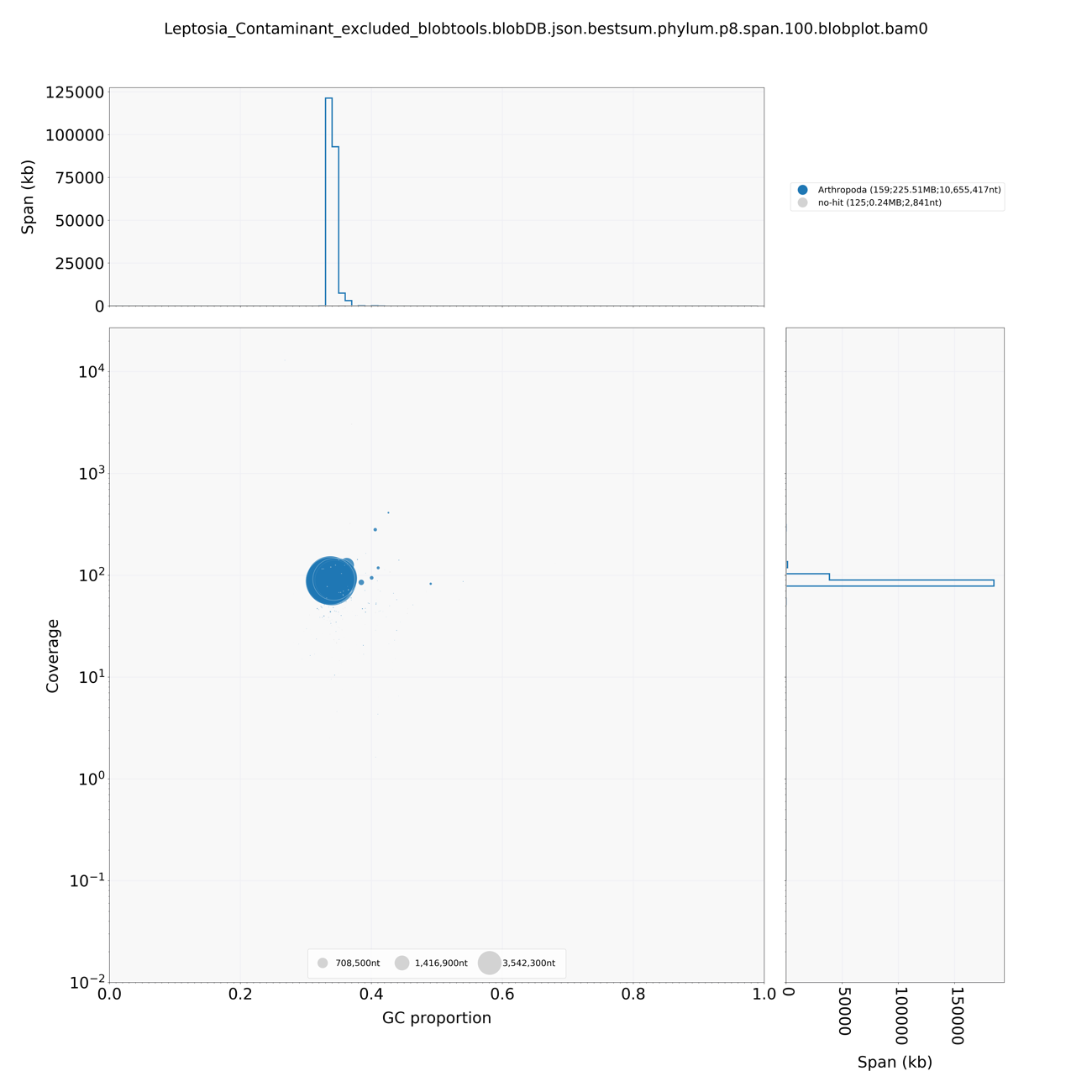


**Fig. S2** A plot based on GC proportion and coverage of each contig generated using blobtools.

**
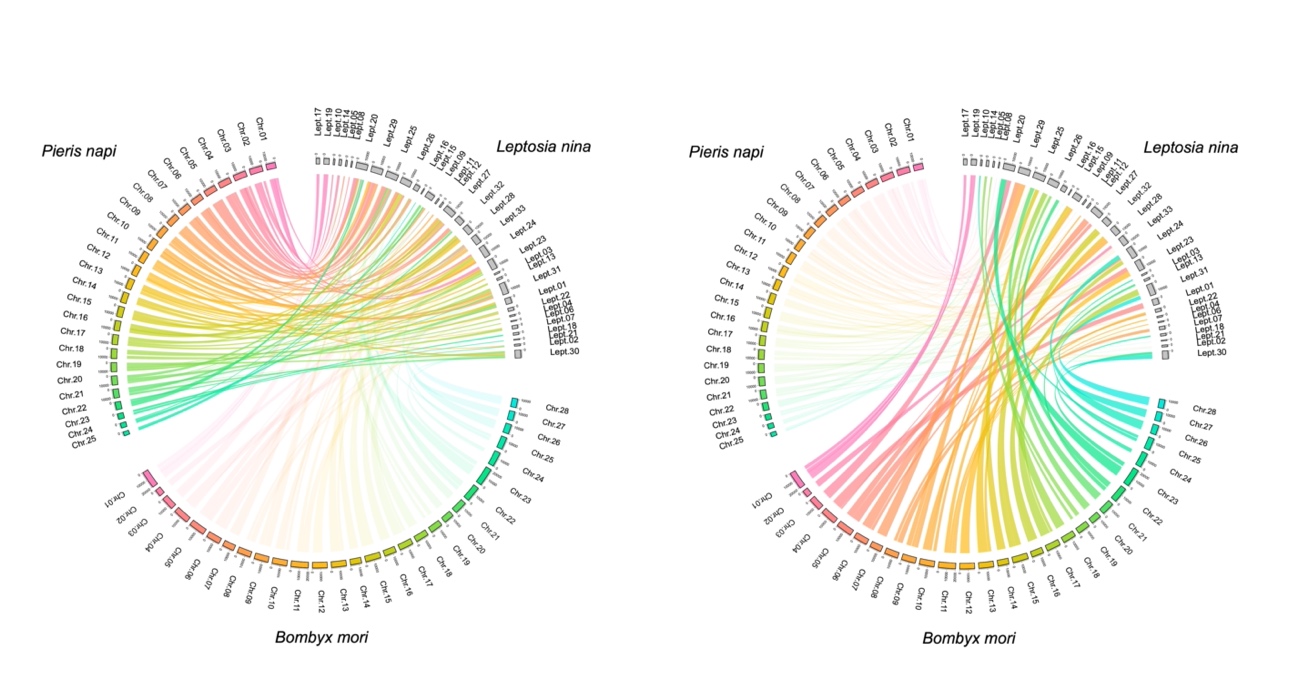
**

**Fig. S3** Synteny plot between the *Leptosia nina*, *Pieris napi*, and *Bombyx mori* genomes.


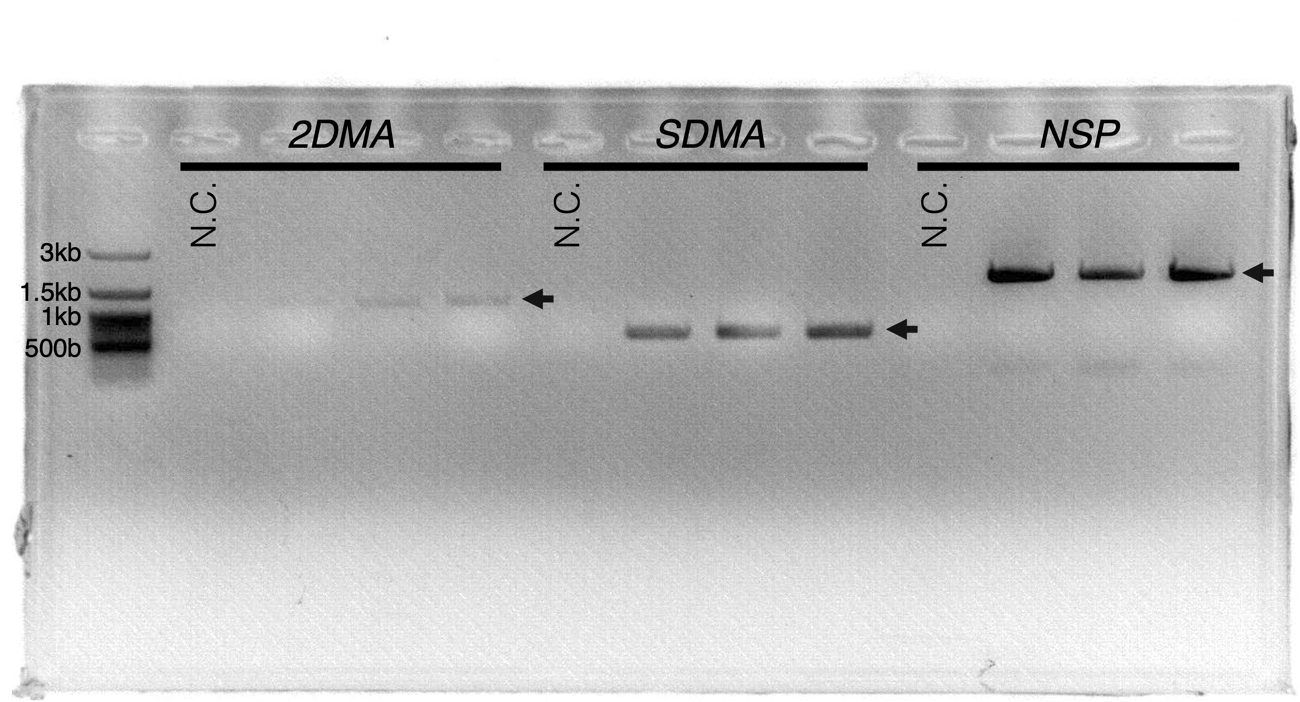

**Fig. S4** The full mRNA PCR products of *2DMA*, *SDMA*, and *NSP* from gut-extracted *Leptosia nina* mRNA (n=3). N.C.: negative control (TotalRNA without cDNA)

**Data S1** Leptosia nina *2DMA* sequence

>Leptosia_2DMA

ATGAAACTTATAATATTGTTGAGTTTTATATGCTTAGGGATGTCGGCGCCCCAGTTGCGCCATTCATTTCAAGATCATTTTGATGACTTCATGATATTGACTCGGGAGTTAGAAGGTGCTCACTTTGGACGACAGACCGAATCATATTTAGGTTTTGAAGAGTTTACTGGAAGCCTTGCTTACCTAGCTAGTAAGGATTTCGTGAAACTGTTTGAAGACTTGAAAGACGTCGCTGAATTCAATGCTGTTCTTGAATTTTTAAAGGACATCGATGTCGATGCATATTACTACCTAGAACTTATTCAACGCTTAGTCGATGAAAAAAGCGCACTGATCACGAAAAAACTACGACACCCAATCACAGGAAGGACAATGACCACATATCTAAATGATATAGTTAGCATGTTCCCCAGAAAAAAATTACGTGAATTGTTCGACGAGAAAATGGATAATGAAAAGGTATTTTCGGGAGTAGTAAAAGAGTTCAGAAGCGAAGAATGGAAAAATCTGTATGAGGCGCTGTGGGAAAATGATAAATTTAAGGCTGTAGTTGATGAGATGGCTGAAAATGATTTCGATCTACGATATGTTTTGGACACAGTGCTGTTGGCTCTCTTTGGACAAATTGAACCGTTTCCGTTAACATTCCAAGACCACTTCGACGATTTTGTTGACACAATTACAGCATTGAAGGGAAAACATTTCGAAGAGCTTGTGAATGAATATCTGCACTTTGAAGAGTTTCAAGCCAGTTTAAATTTTATAGCTGATCAAGATTTTATTGATGTACTCCAAGAATTGATGGAAGTGCCTGCATATGCAACGGCTGATGCATACCTGAAAAGCCATAATATATTCCCTGCTTATTACATCGACCGGTTTCATCTCTTAGCAGAGAAAATACAAGAAAATGCTTCCGGTCAACCCAAGAATTTGCAAGATCTTAAGCACCACCATGCATCTGGACGAACCATGAGGAGTTTTCTTATCGATATAGTTGACATGTTCCCAGAAGAAATATTAGAAGATATTTTAGAAGAGAAAGTAGAGGAGGATACTGAATTCAGAAATGTCTGGAAGAGCCTAAGAAGCGAAGAATGGGAAGACTTATATGAGGCTCTGTGGGAGACTGATATTTTTAAAACCAAAGCTGCTGAGTTAGCTCAATATGATTTTAATTTAGAATACTTTTTCAACACCTTAGTACTGGCTATTTTTGGTCAAGAATGA

**Table S1**The result of branch-model test by PAML on *L. nina* *2DMA*. Null lnL: loglikelihood of a null model (model = 0 was selected in codeml), alternative lnL: loglikelihood of an alternative model (model = 2 was selected in codeml), delta = alternative lnL – null lnL. P-values were obtained from *X*^2^ tests.

| **Gene domain** | **dN/dS** | **null lnL** | **alternative lnL** | **delta** | **p-value** |
| --- | --- | --- | --- | --- | --- |
| Leptosia *2DMA* D1 | 0.0926 | -13406.544 | -13405.667 | 0.876 | 0.186 |
| Leptosia *2DMA* D2 | 0.2496 | -13406.544 | -13406.179 | 0.364 | 0.393 |
| Leptosia *NSP* D1 | 0.0811 | - | - | - | - |
| Leptosia *NSP* D2 | 0.1271 | - | - | - | - |
| Leptosia *NSP* D3 | 0.1046 | - | - | - | - |
| Leptosia *MA* D1 | 0.2614 | - | - | - | - |
| Leptosia *MA* D2 | 0.0950 | - | - | - | - |
| Leptosia *MA* D3 | 0.2057 | - | - | - | - |
| Leptosia *SDMA* | 0.0671 | - | - | - | - |
